# Comparative effectiveness of multiple different non-pharmacologic interventions for post-stroke constipation: a Bayesian network meta-analysis

**DOI:** 10.3389/fneur.2025.1591620

**Published:** 2025-10-10

**Authors:** Sisi Feng, Xinhui Wu, Xuemei Dai, Zhihao Liu, Yi Luo, Fei Wang

**Affiliations:** Chengdu University of Traditional Chinese Medicine, Chengdu, Sichuan, China

**Keywords:** post-stroke constipation, non-pharmacologic interventions, Bayesian network meta-analysis, alternative therapies, rehabilitation

## Abstract

**Background:**

Post-stroke constipation (PSC) is a common complication among stroke patients, with a positive correlation to stroke severity. Straining during defecation in constipated patients can increase intracranial pressure, posing a high risk for secondary strokes, negatively impacting prognosis, disease progression, and contributing to the development of depression and anxiety. Non-pharmacological interventions (NPIs), including traditional Chinese medicine (TCM) and rehabilitation approaches, have been explored due to challenges in advancing Western medical treatments. However, the optimal treatment remains unclear, necessitating guidance for clinical practice. This research employs Bayesian network meta-analysis (NMA) to identify the most effective NPIs for improving clinical outcomes and alleviating constipation in post-stroke patients.

**Methods:**

We conducted a NMA of randomized controlled trials to evaluate the relative efficacy of eight NPIs for PSC: acupuncture therapy (AT), acupoint catgut embedding (ACE), auricular therapy (ART), moxibustion (MT), abdominal massage (AM), point application (PA), physiotherapy (PT), and cognitive behavioral therapy (CBT). The primary outcome was the clinical effective rate (CER), and the secondary outcome was the Constipation Scoring System (CCS). To establish a comparative hierarchy of interventions, surface under the cumulative ranking curve (SUCRA) values were calculated, representing the probability of relative efficacy across treatments.

**Results:**

A comprehensive literature review identified 53 clinical studies with 5,813 participants to evaluate the relative efficacy of eight NPIs. ACE ranked highest for both CER and CCS (SUCRA = 94.7, 97.8%), followed by PT (88.4, 81.7%). In contrast, ART and AM ranked lower, indicating relatively less efficacy compared with other interventions.

**Conclusion:**

Acupoint catgut-embedding (ACE) may represent a potentially superior non-pharmacological intervention for improving clinical outcomes and reducing constipation severity in post-stroke patients. Physiotherapy (PT) also demonstrated favorable efficacy, ranking second in both clinical outcomes. However, further high-quality, multicenter clinical trials are needed to validate and refine these findings.

## Introduction

Post-stroke constipation (PSC) emerges subsequent to an acute cerebrovascular incident, featuring challenges in defecation and dry stools reminiscent of chestnuts. A pervasive complication post-stroke, it manifests universally across stroke types and stages, with an incidence ranging from 30 to 60% (1, 2). Stroke disrupts middle brain nerve conduction, hampering the defecation reflex. Dehydrating agents in initial treatment contribute to dry stools, while prolonged immobility and compromised limb movement decelerate peristalsis. Inadequate dietary fiber intake exacerbates constipation in stroke patients. Conversely, constipated individuals often employ breath-holding, heightening intracranial pressure a high-risk factor for stroke occurrence and potential craniocerebral injury (3). Moreover, prolonged fecal retention facilitates toxin entry into the bloodstream, diminishing nervous system function and impeding neurological recovery in stroke patients (4). This detrimental interplay significantly obstructs stroke rehabilitation, negatively affecting patients’ quality of life and intensifying depression and anxiety. Addressing constipation becomes integral to stroke treatment, emphasizing prevention and symptom alleviation.

In Western medicine, pharmacological interventions (PI) dominate constipation resolution (5, 6). However, these interventions carry numerous adverse effects and are prone to drug resistance. This not only increases patient discomfort but may also protract the disease course. A multinational study by Wald et al. (7), involving over 13,879 questionnaires, revealed persistent constipation in 20–40% of patients despite extensive laxative use. Recognizing the limitations of Western medicine, non-pharmacological interventions (NPIs), such as traditional Chinese medicine (TCM) and rehabilitation interventions, offer more promising avenues. This study comprehensively summarizes the advantages of NPIs for PSC, including acupuncture therapy (AT), acupoint catgut-embedding (ACE), auricular therapy (ART), moxibustion (MT), abdominal massage (AM), point-application (PA), physiotherapy (PT), and cognitive behavioral training (CBT). While advantages vary among these interventions, the absence of guidelines ranking their efficacy for PSC treatment introduces clinical confusion. To address this gap, we propose employing Bayesian network meta-analysis (NMA) to comprehensively analyze eight randomized controlled trials (RCTs) evaluating clinically used NPIs for PSC. Our aim is to identify an optimal protocol for guiding clinical practice, grounded in robust evidence and statistical inference.

## Materials and methods

This study adhered to the Preferred Reporting Items for Systematic Reviews and Meta-Analyses (PRISMA) extended statement guidelines (8). The study is registered under the International Prospective Register of Systematic Reviews (PROSPERO)^1^ with the registration number CRD42022377376. Given that all analyses were built upon previously published research, ethical approval and patient consent were deemed unnecessary for this investigation.

### Search strategy

The comprehensive search encompassed multiple databases, including Web of Science, PubMed, EMBASE, Cochrane Central Controlled Trials, China Knowledge Network (CNKI), Wanfang database, VIP database, and China Biomedical Literature Database (CBM). The search targeted RCTs assessing the effectiveness of NPIs in PSC up to September 6, 2025. Language limitations were not imposed.

Utilizing a meticulous approach, the search strategy integrated Medical Subject Headings (MeSH) and free words, employing Boolean logic operators. The key terms incorporated for the comprehensive search were “stroke,” “cerebral infarction,” “cerebral hemorrhage,” “constipation,” “dyschezia,” “non-pharmacological interventions,” “acupuncture therapy,” “acupoint catgut-embedding,” “auricular therapy,” “moxibustion,” “abdominal massage,” “point-application,” “physiotherapy,” “rehabilitation,” “cognitive behavioral training,” and “randomized controlled trial.” To further enhance inclusivity, a manual review of reference lists in relevant meta-analyses and reviews was conducted, mitigating the risk of excluding literature meeting inclusion criteria. Specific details of the search strategy, exemplified by the PUBMED search, are provided in Supplementary Table 1. Two independent authors, Xinhui Wu and Xuemei Dai, conducted the screening using Endnote 20 literature management software (Thompson ISI Research Soft, Philadelphia, PA, USA). Any discrepancies during this process were resolved through consensus or consultation with a third author, Wang Fei, ensuring the reliability and transparency of the literature selection process.

### Selection and exclusion criteria

Inclusion criteria: The study selection strictly followed the PICOS framework.

(1) Population: Patients diagnosed with PSC based on established diagnostic criteria (9, 10), without restrictions on age or gender.
(2) Intervention: Studies evaluating one of the eight predefined NPIs (AT, ACE, ART, MT, AM, PA, PT, CBT). Interventions had to be clearly described with adequate details on frequency, duration, or modality.
(3) Comparison: Eligible comparators included PI, placebo (routine dietary care alone), or comparisons between NPIs.
(4) Outcomes: The primary outcome was the Clinical Effective Rate (CER). Secondary outcomes included the Cleveland Clinic Score (CCS). Studies had to report at least one of these outcomes.
(5) Study design: Only randomized controlled trials (RCTs) were eligible.

Exclusion criteria:

(1) Non-RCTs, including observational studies (cohort, case–control, cross-sectional), reviews, systematic reviews, case reports/series, and study protocols.
(2) Animal experiments or *in vitro* studies.
(3) Studies without full-text availability (e.g., conference abstracts).
(4) Duplicate publications from the same trial; in such cases, the report with the most complete data was retained.
(5) Studies with insufficient or missing outcome data that could not be obtained after contacting the authors.
(6) Interventions with poorly defined content, frequency, or intensity.
(7) Comparisons not involving PI, placebo, or other NPIs.

Utilizing these criteria, two authors (X.W. and X.D.) independently assessed titles and abstracts, eliminating duplicate titles and studies not meeting inclusion criteria. Subsequently, the studies meeting the criteria underwent thorough examination. Any discrepancies during this process were resolved through consensus, ensuring a rigorous and systematic study selection process.

### Data extraction and quality assessment

Relevant information from eligible studies was systematically gathered utilizing the Cochrane Consumer and Communications Review Group’s data extraction template. This comprehensive data collection encompassed essential publication details, participant characteristics (total sample size, age, and disease duration), intervention specifics, treatment duration, and the quality assessment of RCTs, along with other pertinent data.

Two independent researchers, ZHL and YL, rigorously assessed the quality of each eligible study utilizing the Cochrane Risk of Bias Tool (11). This tool, applied across seven domains, facilitated a thorough evaluation of each project’s risk of bias, categorized as unknown, low, or high. The quality assessment process was executed using Review Manager (version 5.4), ensuring a standardized and reliable evaluation of study quality. Any discrepancies during the assessment were resolved through consensus, maintaining the integrity and robustness of the quality evaluation process.

### Statistical analyses

Utilizing minimally informative prior distributions in the Bayesian random effects model (12), we initially conducted a conventional pair-wise meta-analysis, amalgamating crucial data from all included studies. Effect sizes were computed using odds ratios (OR), and corresponding 95% credible intervals (CrIs) quantified group effects. Mean differences (MD) with 95% CrIs were employed to assess estimated and pooled effect sizes. Statistical heterogeneity was visually represented using the *I*^2^ statistic to identify significant heterogeneity (13). To identify potential bias, a comparison-adjusted funnel plot was constructed, followed by quadratic validation using the Egger’s test (14). A network plot was generated to visually represent existing relationships, with different treatments as nodes and trials as connecting lines.

The evaluation of network transitivity is pivotal in NMA and significantly influences subsequent analyses (15). To ensure the comparability of different treatments and the validity of drawing indirect conclusions, we scrutinized the transitivity assumption. This involved a meticulous comparison of clinical and methodological characteristics, including participant attributes and experimental design, across all included studies (16, 17). For precise estimation of the statistical model, we established four parallel Markov chains in the random selection state (18). Each chain underwent 20,000 iterations, with an initial burn-in period discarding the first 5,000 iterations. This practice minimized bias from initial values when the chain reached its target distribution (19). Convergence evaluation employed the Brooks-Gelman-Rubin diagnostic, involving a visual inspection of the historical trajectory of trace combined with density plots (20) (Supplementary Figure 1). The Surface Under the Cumulative Ranking Curve (SUCRA) served as a hierarchical tool for ranking the efficacy of various NPIs for PSC. Utilized as an ordinary numerical statistic cumulative ranking probability diagram, SUCRA summed up each treatment. Higher SUCRA values suggested that a presented treatment could be at the top level or highly valid for PSC patients, while a value of 0 indicated the least effective treatment (21). To explore potential source inconsistency in our network, we employed the “node splitting” technique, comparing direct and indirect evidence throughout the network. Consistency was considered to arise when *p* > 0.05 (22, 23). All analyses were conducted using the “Gemtc” package (version 1.0–1) and “rjags” (version 4–13) in R software (4.1.3 version), and STATA Version 16.0 (StataCorp, College Station, TX, USA).

Importantly, the methodological framework for this NMA was grounded in our previously published work (24). Building on the robust foundation established in that study, we carefully refined and adapted the evaluation processes to align with the specific objectives and scope of the current analysis. This approach ensured methodological rigor, precision, and consistency throughout the study.

## Results

### Search process and baseline characteristics

**Figure 1:**
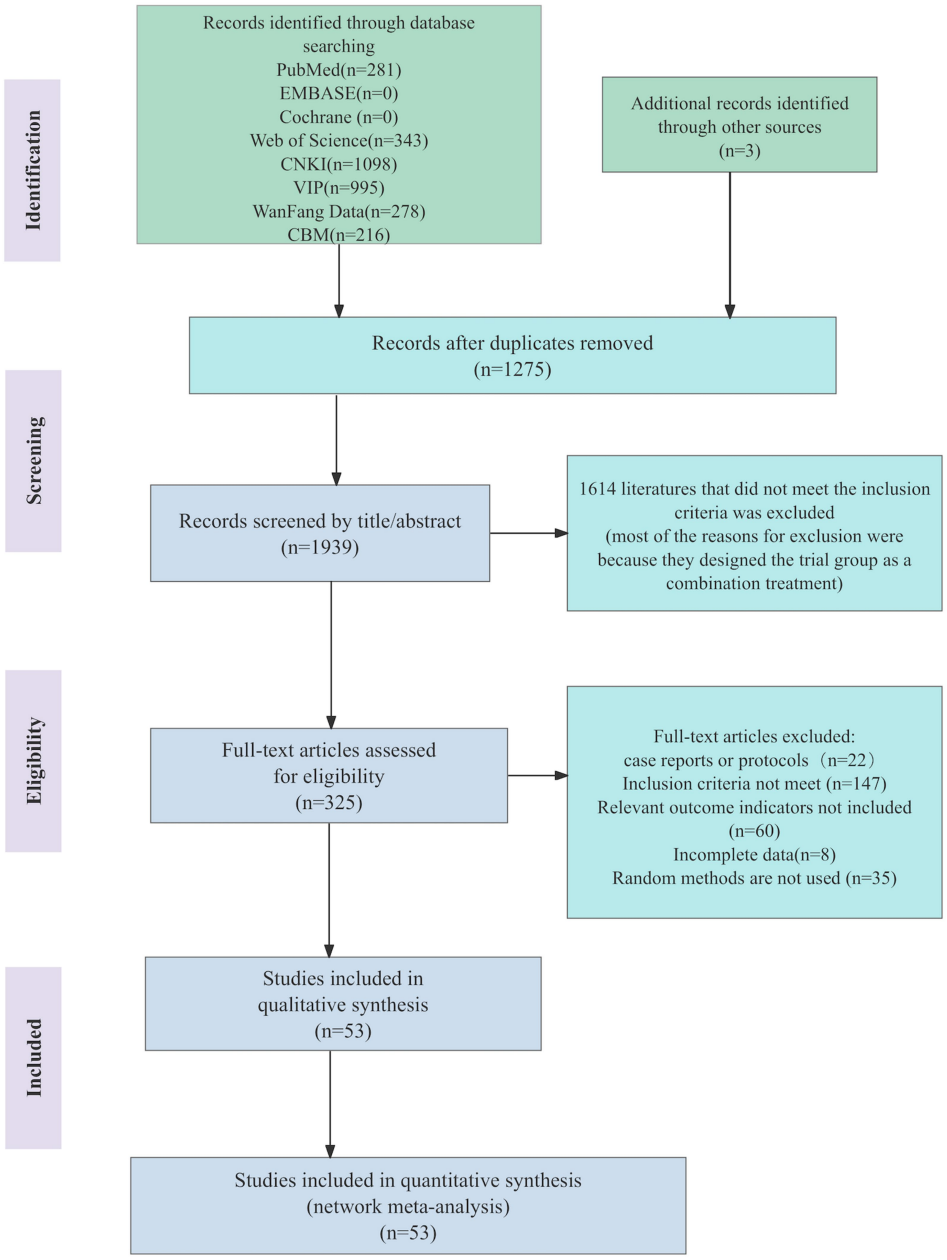
Literature screening process.

The search initially yielded 3,214 pieces of literature, with 1,275 duplicates identified. Upon title and abstract assessment, 1,614 documents were deemed ineligible and subsequently excluded. A comprehensive analysis of the remaining 325 studies resulted in the exclusion of 147 studies for not meeting inclusion criteria. Additionally, 60 studies lacked relevant outcome indicators, 22 were case reports or study protocols, 35 did not mention the specific method of randomization, and 8 were either unavailable in full text or had incomplete outcome indicators. Consequently, 53 RCTs meeting the specified criteria were included in the analysis (25–77). Figure 1 illustrates the process of literature screening.

Table 1 presents a comprehensive overview of essential features, including participant baseline characteristics and interventions. All included studies were from China and published between 2010 and 2024. A total of 3,904 participants were randomly assigned to trial or control groups. Among them, 1,909 participants were in the trial group, undergoing eight different NPIs (AT, *n* = 384; ACE, *n* = 216; MT, *n* = 276; AM, *n* = 122; PA, *n* = 289; ART, *n* = 190; PT, *n* = 187; CBT, *n* = 245). The remaining 1,995 individuals were randomized into five control groups (PI, *n* = 1,044; Placebo, *n* = 532; AT, *n* = 294; CBT, *n* = 50; AM, *n* = 75).

**Table 1:** Characteristic of included studies.

Study ID	Participant			Age	Gender (M/F)	Interventions			Course	Outcome
	T	C1	C2			T	C1	C2		
Ma, 2023 (25)	30	30		T: 63.00 ± 8.00 C: 66.00 ± 8.00	T: 22/8 C: 20/10	MT	AT		2w	CER CCS
Chen, 2022 (26)	54	56		T: 67.10 ± 3.40 C: 65.60 ± 3.20	T: 37/17 C: 33/23	AT	placebo		4w	CER CCS
Zhong, 2022 (27)	30	30		T: 59.73 ± 4.75 C: 60.40 ± 4.39	T: 16/14 C: 14/16	AT	PI		4w	CER CCS
Wang, 2022 (28)	45	45		T: 59.93 ± 9.06 C: 61.80 ± 8.28	T: 24/21 C: 22/23	PA	AM		7d	CER CCS
Zhang, 2022 (29)	30	31		T: 57.07 ± 7.65 C: 54.23 ± 7.43	T: 19/11 C: 17/14	PA	placebo		2w	CER CCS
Yuan, 2021 (30)	32	32		T: 60.63 ± 6.74 C: 63.19 ± 7.47	T: 25/7 C: 23/9	AT	PI		2w	CER
Xue, 2021 (31)	52	51		T: 58.12 ± 4.36 C:58.44 ± 4.08	T: 29/23 C: 31/20	AT	placebo		2w	CER
Wang, 2021 (32)	37	37		T:52.78 ± 10.28 C:55.03 ± 11.29	T: 23/14 C: 21/16	AT	PI		10d	CER
He, 2021 (33)	42	42		T: 72.26 ± 12.36 C: 71.86 ± 9.65	T: 25/17 C: 20/22	PT	PI		7d	CER
Huang, 2021 (34)	30	30		T: 69.57 ± 2.57 C: 70.17 ± 2.59	T: 15/15 C: 16/ 14	CBT	placebo		4w	CER CCS
Song, 2021 (35)	40	40		T: 67.31 ± 9.03 C: 71.09 ± 7.80	T: 26/14 C: 23/17	PA	PI		7d	CCS
Gao, 2020 (36)	30	30		T: 64.53 ± 6.10 C: 65.00 ± 6.87	T: 14/16 C: 17/13	ACE	AT		3w	CER
Du, 2020 (37)	50	50		T: 69.53 ± 7.29 C: 68.94 ± 7.26	T: 29/21 C: 28/22	PT	CBT		2w	CCS
Cong, 2020 (38)	24	29		T: 59.75 ± 11.25 C:58.64 ± 10.34	T: 17/7 C: 20/9	CBT	placebo		2w	CER CCS
Liang, 2020 (39)	50	50		T: 64.13 ± 8.80 C: 63.80 ± 7.82	T: 21/29 C: 31/19	AM	PI		7d	CER
Zhang, 2019 (40)	30	30		T: 57.43 ± 10.23 C:58.47 ± 10.29	T: 13/17 C: 17/13	MT	AT		2w	CER CCS
Sun, 2019 (41)	30	30		T: 63.23 ± 5.67 C: 64.30 ± 6.18	T: 17/13 C: 18/12	ACE	PI		4w	CCS
Guo, 2019 (42)	35	34		T: 56.83 ± 8.27 C: 56.18 ± 8.00	T: 20/15 C: 20/14	ACE	AT		4w	CER CCS
Wu, 2019 (43)	23	23		T: 62.26 ± 8.48 C: 61.60 ± 7.75	T: 13/10 C: 14/9	AT	PI		2w	CCS
Deng, 2019 (44)	29	29		T: 60.57 ± 1.37 C: 61.25 ± 1.23	T: 19/10 C: 18/11	ACE	AT		4w	CER
Huang, 2019 (45)	30	29		T: 61.89 ± 3.85 C: 62.28 ± 3.54	/	ACE	AT		2w	CER CCS
Yu, 2017 (53)	15	13		T: 62.60 ± 10.0 C:61.30 ± 11.22	T: 10/5 C: 5/8	PA	placebo		7d	CCS
Liu, 2016 (54)	30	30		T: 61.03 ± 9.50 C: 60.97 ± 9.77	T: 18/12 C: 15/15	MT	PI		2w	CCS
Peng, 2016 (55)	24	24		T: 51.00 ± 6.00 C: 53.00 ± 7.00	T: 10/14 C: 12/12	AT	PI		2w	CER CCS
Ji, 2016 (56)	40	40		T: 61.00 ± 9.00 C: 60.00 ± 10.0	T: 19/21 C: 26/14	ART	PI		2w	CER CCS
Huang, 2014 (68)	48	48		T: 61.82 ± 9.13 C: 60.57 ± 9.76	T: 35/13 C: 30/18	CET	PI		2w	CCS

### Quality of included studies

The risk of bias graph and summary are detailed in Supplementary Figure 2. All 53 studies (25–77) utilized group randomization and provided comprehensive data on predetermined outcome measures. Twelve studies (30, 31, 36, 45, 53, 62–65, 67, 72, 77) explicitly stated the use of allocation concealment, three studies (46, 52, 54) implemented participant blinding, seven studies (29, 41, 53, 54, 59, 63) employed blinding to evaluate outcomes, 16 studies (27, 29–32, 36, 42, 43, 45, 46, 50, 54, 56, 62–64) had late follow-up, and 16 studies (27, 31, 32, 34, 36, 41, 45, 49, 53, 63, 64, 67, 71, 72, 75, 76) provided information on case disengagement or adverse events, while the exact implementation of other studies is unclear.

### Network analysis results

#### Primary outcome: CER

The preliminary conventional meta-analysis (*I*^2^ = 0%, *p* = 0.000) showed negligible heterogeneity. The adjusted funnel plot showed an approximately symmetrical distribution, with a few studies scattered at the bottom suggesting potential publication bias (Supplementary Figure 3). However, additional Egger’s test (*p* = 0.086 (>0.05)) confirmed no publication bias in the CER outcome (Supplementary Table 2).

**Figure 2:**
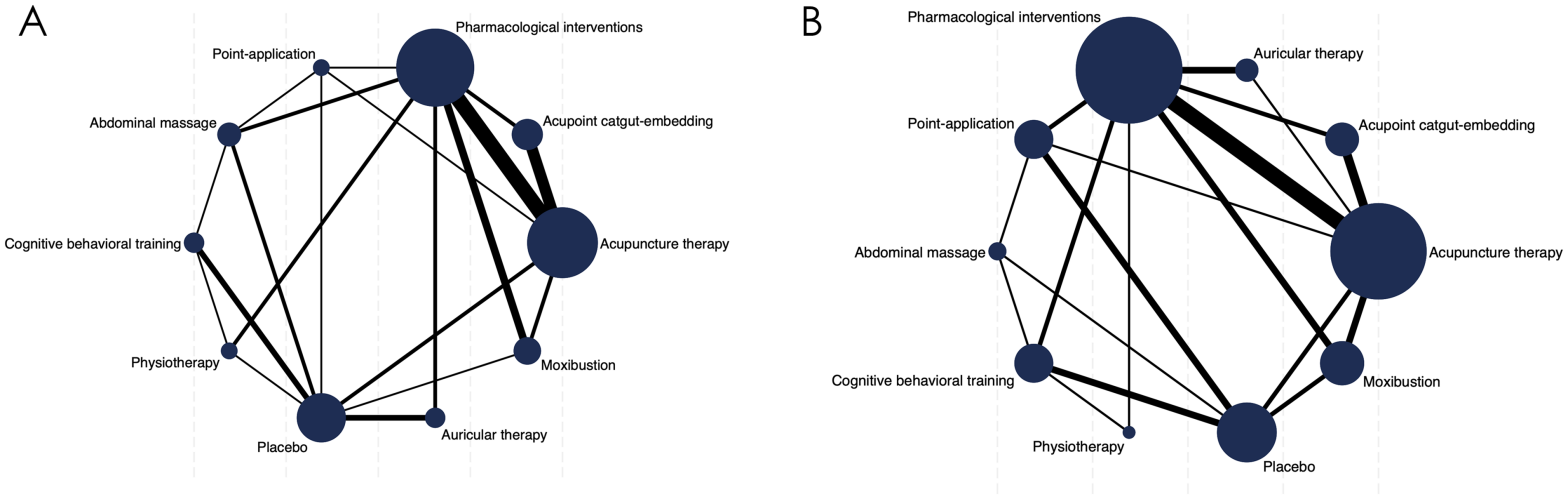
The network evidence graph. **(A)** Clinical Effective Rate (CER); **(B)** Cleveland Clinic Score (CCS).

NPIs and two control groups (pharmacological interventions (PI) and placebo) are depicted in the network diagram (Figure 2). Each node represents a specific intervention, with node size proportional to the number of patients included. AT was the most extensively studied, with 9 arms (*n* = 660), followed by ACE and MT, each evaluated in 7 arms (*n* = 460 and *n* = 436, respectively), and ART (*n* = 339) in 5 arms. PT and PA were investigated in 4 arms each (*n* = 374 and *n* = 397), while CBT (*n* = 244) and AM (*n* = 203) were the least studied, with only 3 arms.

**Figure 3:**
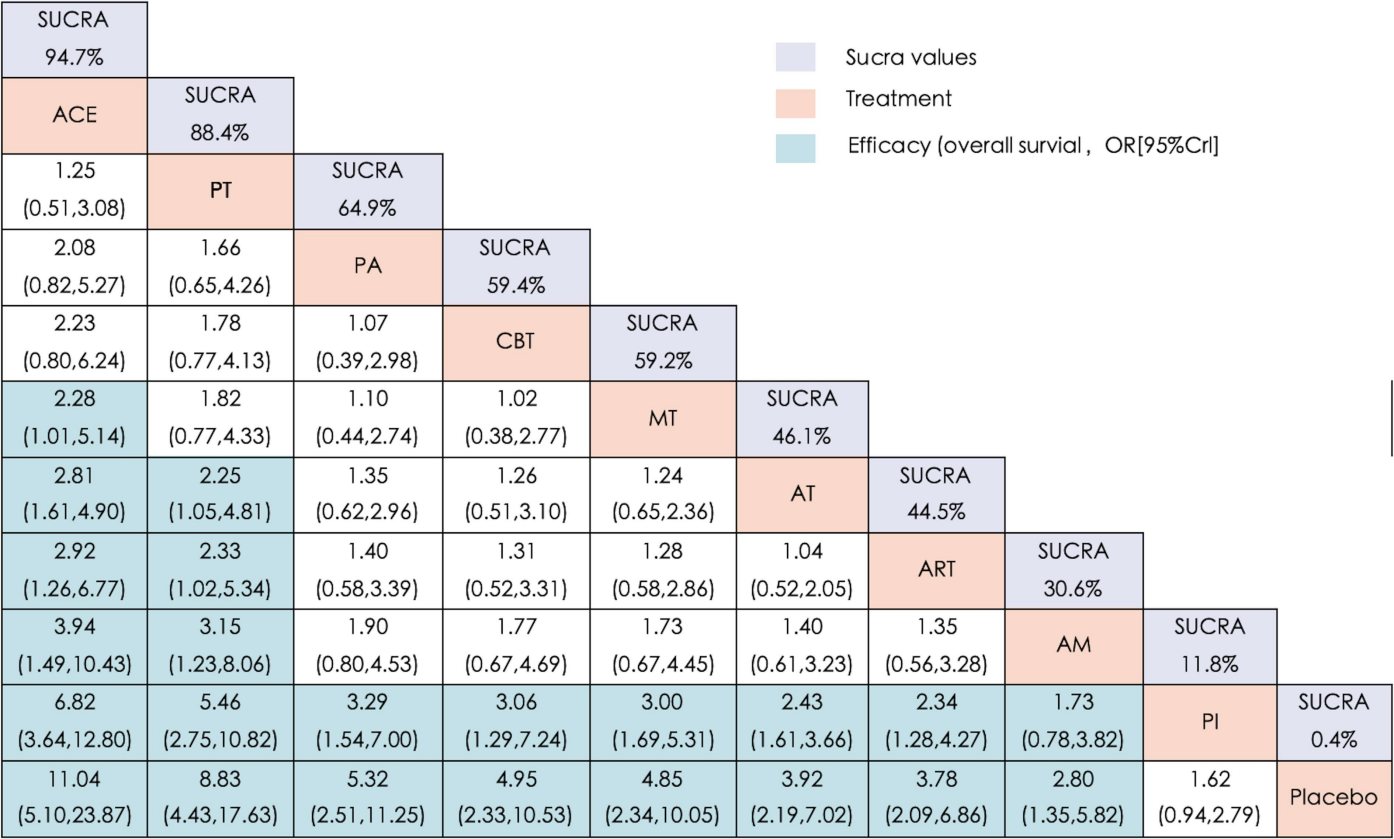
League matrix table of CER. Treatments were ranked in order of their likelihood of being the best treatment. The numbers in the purple boxes are SUCRA values, representing the rank of the treatments. Meaningful pairwise comparisons are shown in blue boxes. ACE, Acupoint Catgut-embedding; PT, Physiotherapy; MT, Moxibustion; PA, Point-application; AT, Acupuncture Therapy; ART, Auricular Therapy; AM, Abdominal Massage; CBT, Cognitive Behavioral Training; PI, Pharmacological Interventions.

Figure 3 summarizes the comparative effectiveness of NPIs on the CER outcome. All eight interventions were associated with significantly improved outcomes compared with the control group, except that no significant difference was observed between AM and PI. Both ACE and PT appeared more effective than AT (OR = 2.92, 95% CrI: 1.26–6.77; OR = 2.33, 95% CrI: 1.02–5.34), ART (OR = 2.21, 95% CrI: 1.07–4.57; OR = 2.14, 95% CrI: 1.01–4.54), and AM (OR = 3.94, 95% CrI: 1.49–10.43; OR = 3.15, 95% CrI: 1.23–8.06). In addition, ACE was associated with significantly greater efficacy than MT (OR = 2.28, 95% CrI: 1.01–5.14). Overall, the findings suggest that ACE and PT may provide more consistent advantages compared with several other interventions in improving CER outcomes.

**Figure 4:**
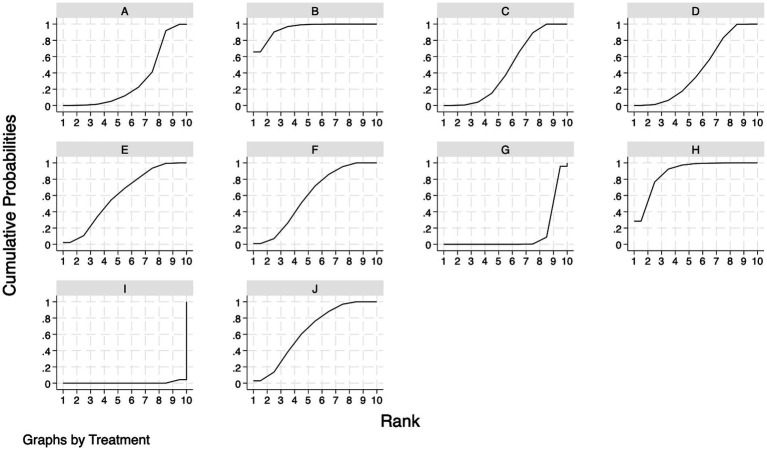
Probability ranking results of different interventions. **(A)** Abdominal massage; **(B)** Acupoint catgut-embedding, **(C)** Acupuncture therapy; **(D)** Auricular therapy; **(E)** Cognitive behavioral training; **(F)** Moxibustion; **(G)** Pharmacological interventions; **(H)** Physiotherapy; **(I)** Placebo; **(J)** Point-application.

The SUCRA rankings for each intervention are shown in Figures 3, 4. ACE had the highest probability of improving CER in patients with PSC (94.7%), followed by PT (88.4%). PA (64.9%), CBT (59.4%), and MT (59.2%) occupied the next tier with comparable probabilities. AT (46.1%) ranked sixth, while ART (44.5%) and AM (30.6%) were seventh and eighth, respectively. PI (11.8%) and placebo (0.4%) were lowest. The “nodal split” method was employed to assess inconsistencies between direct and indirect evidence. Results (Supplementary Figure 4) indicated no significant inconsistencies in the network’s branches (*p* > 0.05). However, notable inconsistencies were observed in local inconsistency tests for (E-A) (*p* = 0.02), (I-A) (*p* = 0.035), (I-E) (*p* = 0.023), and (I-F) (*p* = 0.0035) suggesting some inconsistency in this closed loop.

#### Secondary outcome: CCS

The preliminary meta-analysis revealed substantial heterogeneity in CCS outcomes across the included studies (*I*^2^ = 84.2%, *p* < 0.001), therefore, a random-effects model was employed to account for the high between-study variability. The funnel plot for the secondary outcome showed mild asymmetry, characterized by an over-representation of studies on the right side and a scattering of points at the bottom, suggesting the possibility of publication bias or underlying heterogeneity (Supplementary Figure 3). Nevertheless, Egger’s test did not detect significant publication bias (*p* = 0.732, Supplementary Table 3). To further investigate potential sources of heterogeneity, we performed sensitivity analyses and meta-regression, as presented in the ‘Sensitivity Analysis and Meta-Regression’ section of the Results.

A network diagram is presented in Figure 2, comprising seven active interventions and three control groups (PI, placebo, and AM, which was treated as a control in this outcome measure). Among the interventions, MT (*n* = 486) and AT (*n* = 545) were the most extensively evaluated, each included in eight study arms, followed by PA (*n* = 580) with seven arms. ACE (*n* = 342) and CBT (*n* = 405) were assessed in five arms, whereas PT (*n* = 230) and ART (*n* = 120) were the least studied, with only two arms each.

**Figure 5:**
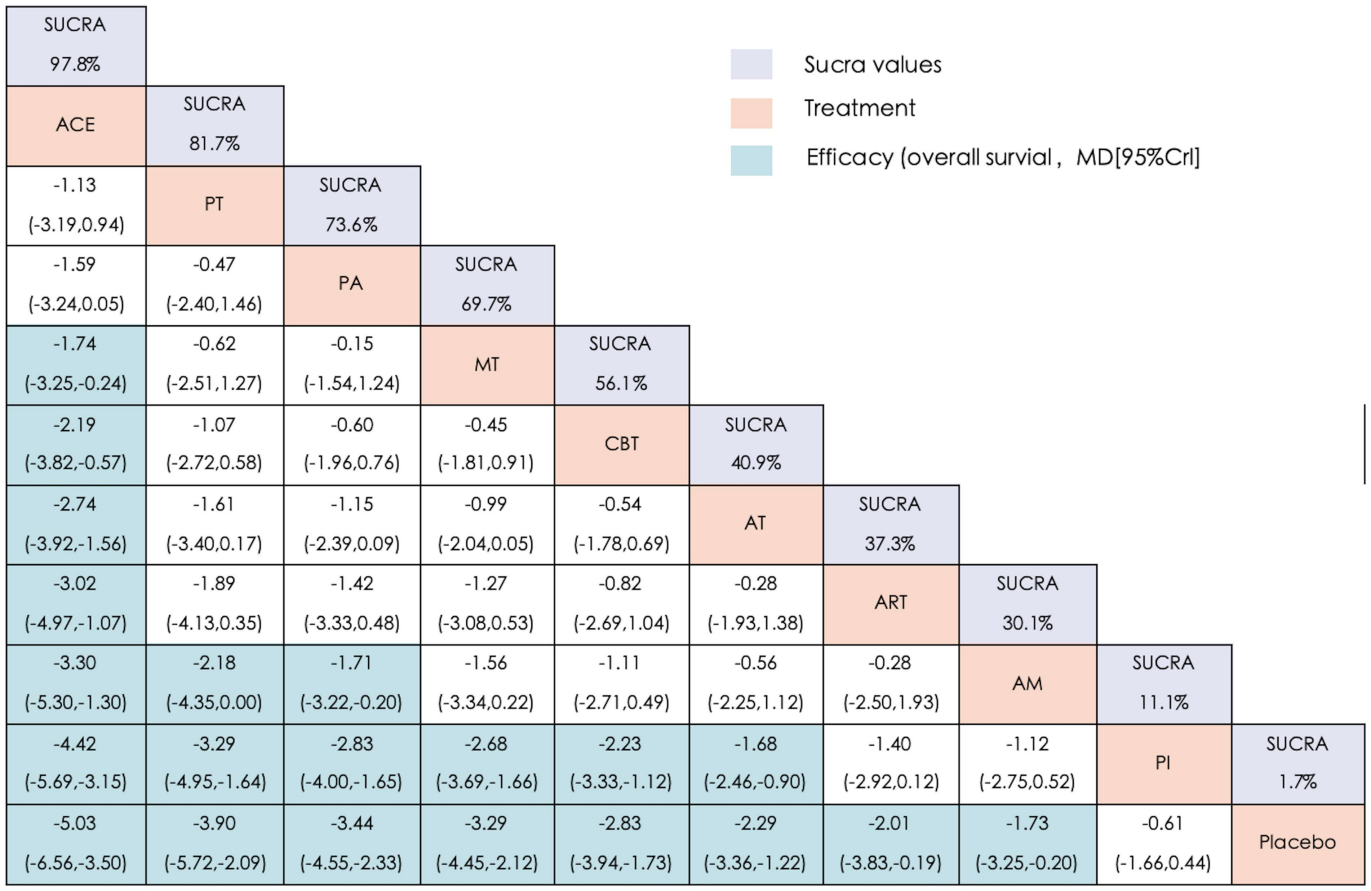
League matrix table of CCS. Treatments were ranked in order of their likelihood of being the best treatment. The numbers in the purple boxes are SUCRA values, representing the rank of the treatments. Meaningful pairwise comparisons are shown in blue boxes. ACE, Acupoint Catgut-embedding; PT, Physiotherapy; MT, Moxibustion; PA, Point-application; AT, Acupuncture Therapy; ART, Auricular Therapy; AM, Abdominal Massage; CBT, Cognitive Behavioral Training; PI, Pharmacological Interventions.

The efficacy outcomes for constipation improvement are summarized in Figure 5. Compared with the three control groups (AM, PI, and placebo), ACE, PT, and PA were associated with significant improvements. ACE also demonstrated superiority over MT (MD = −1.74, 95% CrI: −3.25, −0.24), CBT (MD = −2.19, 95% CrI: −3.82, −0.57), AT (MD = −2.74, 95% CrI: −3.92, −1.56) and ART (MD = −3.02, 95% CrI: −4.97, −1.07), ranking highest among all interventions. Notably, no significant differences were observed between ART and either PI or AM.

The SUCRA rankings for CCS (Figures 4, 5) were largely consistent with those for the primary outcome (CER), with the following hierarchy: ACE (97.8%) > PT (81.7%) > PA (73.6%) > MT (69.7%) > CBT (56.1%) > AT (40.9%) > ART (37.3%) > AM (30.1%) > PI (11.1%) > placebo (1.7%). Furthermore, Supplementary Figure 5 illustrates that the node segmentation model results still indicate some inconsistency in direct and indirect evidence between individual studies.

### Sensitivity analysis and meta regression

In the sensitivity analysis (Supplementary Figure 6), we aimed to explore the source of heterogeneity in the CCS results. Consistent with the preliminary analysis, excluding any single study had minimal impact on the composite results, suggesting that the composite effect sizes in this study remain relatively stable. Additionally, we conducted a more detailed subgroup analysis by categorizing the 35 studies into four groups for meta-regression analyses based on the year of publication year, intervention time, and control objects (Supplementary Table 4). The results indicated that none of the four study groups significantly contributed to the generation of heterogeneity (*p* > 0.05).

## Discussion

Existing clinical trials have predominantly assessed the relative efficacy of individual non-pharmacological interventions (NPIs) in managing post-stroke constipation (PSC). Traditional meta-analyses have often been limited to evaluating the effectiveness of singular interventions. However, the introduction of network meta-analysis (NMA) has addressed this limitation by integrating two or more interventions. NMA enables both direct and indirect cross-comparisons, adjusting for indirect comparisons and simultaneously evaluating and ranking all included interventions (78). This study is the first to apply the NMA approach to compare the efficacy of various NPIs in PSC patients. Its complex and comprehensive methodology surpasses the majority of previous studies, providing a foundation for evidence-based clinical guidelines that can guide the selection of optimal treatment regimens for PSC in the future.

PSC hampers recovery and increases the risk of secondary complications. While pharmacological treatments and enemas (e.g., commonly used agents such as lactulose, polyethylene glycol, or traditional Chinese herbal cathartic preparations like rhubarb-based formulas) can relieve symptoms, prolonged use can lead to adverse effects, dependence, and colonic dysfunction (79). These limitations, along with common challenges associated with PSC (such as frailty, dysphagia, and limited mobility), have driven a shift toward non-pharmacological management. Traditional Chinese medicine (TCM) NPIs and modern rehabilitation techniques (e.g., physiotherapy, PT) have shown positive effects and are generally better tolerated by patients, making them a rational focus for evaluation. Accordingly, we conducted a systematic review of eight NPIs, including acupuncture therapy (AT), acupoint catgut-embedding (ACE), auricular therapy (ART), moxibustion (MT), abdominal massage (AM), point-application (PA), physiotherapy (PT), and cognitive behavioral training (CBT), to clarify their comparative advantages and clinical applicability. Our findings indicate that ACE was ranked highest for both clinical efficacy (SUCRA = 94.7%) and alleviating constipation severity as measured by the constipation severity scale (CCS) (98.2%). PT (88.4, 82.0%) and PA (64.9, 73.0%) followed, with ART and AM ranked relatively lower.

Acupoint catgut-embedding (ACE) is an advancement of traditional acupuncture therapy (AT), which involves the implantation of absorbable sheep intestinal sutures into specific acupoints. Once embedded, the suture material gradually softens and decomposes, providing continuous stimulation that can last for at least 15 days—an effect that differs from conventional needling or burying techniques (80, 81). The therapeutic action of ACE combines several mechanisms, including acupoint blockade, AT stimulation, blood-letting effects, prolonged needle action, and mild tissue injury, thus delivering a multimodal stimulus (82, 83). Experimental studies have shown that ACE elicits various biochemical responses and bidirectional regulation at acupuncture points, peripheral nerves, and the central nervous system (84, 85). This stimulation activates parasympathetic pathways, promoting sustained neuromuscular repair of anorectal function, enhancing intestinal peristalsis, and modulating anal sphincter contraction. Simultaneously, ACE downregulates sympathetic activity, increases the sensitivity of anal sphincter pressure receptors, and facilitates the recovery of the defecation reflex. Together, these effects contribute to the normalization of gastrointestinal electrical rhythms and the restoration of regular bowel movements in constipated patients.

In this study, ACE was ranked first in both CER and CCS. Specifically, CCS results revealed that ACE also demonstrated superiority over MT (MD = −1.74, 95% CrI: −3.25, −0.24), CBT (MD = −2.19, 95% CrI: −3.82, −0.57), AT (MD = −2.74, 95% CrI: −3.92, −1.56) and ART (MD = −3.02, 95% CrI: −4.97, −1.07), ranked highest among all evaluated interventions. Although AT has been shown to be beneficial in managing PSC, particularly by reducing laxative use and related adverse effects (86), its clinical application is often hindered by practical limitations, such as the need for prolonged fixed positioning during needle retention and frequent therapeutic sessions. These factors may increase patient discomfort and resistance. In contrast, ACE offers a less burdensome procedure with longer treatment intervals, broader efficacy, and more sustained therapeutic effects, making it particularly advantageous for stroke patients. Moreover, ACE’s safety profile has been consistently confirmed in related clinical studies (87).

Next, physiotherapy (PT) was ranked second across both outcome measures (Clinical efficacy SUCRA = 88.4%; CCS SUCRA = 81.7%), demonstrating favorable efficacy. PT for PSC typically involves electrotherapy, such as medium-frequency pulsed stimulation or transcutaneous acupoint electrical stimulation. As an emerging technique derived from modern medical research, electrotherapy is non-invasive, comfortable, and well-accepted by patients. Medium-frequency electrical stimulation produces therapeutic effects by delivering modulated waveforms and amplitudes to relevant acupoints. This generates mechanical-like effects (e.g., pushing, pressing, squeezing) that facilitate the transmission of stimulation to the rectum and anus, activate the sacral nerve and pelvic plexus, and enhance rectal afferent excitability. This process improves local blood circulation, augments intestinal peristalsis, and contributes to regulating disrupted intestinal flora and neurotransmitter levels (88, 89). Transcutaneous acupoint electrical stimulation, an evolution of traditional acupuncture, uses parameterized electrical devices affixed to acupoints to achieve therapeutic outcomes similar to acupuncture without invasive manipulation (90, 91). Stimulation signals are transmitted through afferent nerves innervating local skin and muscle at acupuncture points, integrated in the spinal cord and supraspinal centers (e.g., vagal complex, hypothalamus), and ultimately conveyed through sympathetic and parasympathetic pathways. These outputs regulate gut function and alleviate constipation, engaging the brain-gut axis mechanism (92).

As highlighted in studies on PSC pathogenesis (93), stroke-induced injury to the cortical defecation center disrupts autonomic reflexes, impairing defecation and reducing intestinal motility. The brain-gut axis, a neuroendocrine network linking the gastrointestinal tract and central nervous system (CNS), plays a crucial role in this process. Brain-gut peptides enable the CNS to transmit regulatory impulses to the gastrointestinal tract, while the enteric nervous system (ENS) exerts feedback regulation on the CNS. This bidirectional modulation of gastrointestinal function underpins the therapeutic rationale for PT in PSC (94).

Point-application (PA) ranked third overall, showing superiority over pharmacological interventions and placebo but remaining less effective than ACE and PT. Rooted in TCM theory, PA combines herbal formulations with transdermal delivery at specific acupoints, achieving synergistic effects between drug absorption and acupoint stimulation (95, 96). Its advantages include avoiding hepatic first-pass metabolism, prolonged release of active ingredients, and good patient compliance (97, 98). However, variability in herbal selection, lack of standardized protocols, and limited high-quality clinical evidence limit its broader clinical adoption. While PA may serve as a complementary non-pharmacological option for PSC, particularly in patients unsuitable for invasive therapies, further rigorously designed trials are needed to establish its efficacy and optimize treatment protocols.

## Limitations

While this investigation provides valuable insights into NPIs for PSC, several limitations should be acknowledged. First, the reliance on aggregate-level comparisons may have overlooked heterogeneity introduced by confounding factors, such as the choice of acupuncture points and the frequency of electrical stimulation. Second, the lack of AM trials included as comparators for the CCS outcome may have introduced potential bias into the analysis. Third, the concentration of available studies in specific regions may limit the generalizability of our findings to broader populations. Future research with more geographically diverse samples and refined intervention protocols will be essential to establish a more comprehensive understanding of NPIs for PSC.

## Conclusion

In conclusion, this study synthesized evidence from eight trials. While various NPIs demonstrated different degrees of symptom improvement in PSC, the NMA findings indicate that ACE may be associated with comparatively greater benefits for clinical efficacy and constipation severity. PT and PA also showed potential advantages and could be considered as alternative approaches. In contrast, ART and AM appeared less effective; however, the overall certainty of evidence remains limited. Taken together, these findings should be interpreted with caution and regarded as preliminary insights to guide clinical decision-making, underscoring the need for further high-quality research to validate these observations.

Although the preliminary analysis revealed considerable statistical heterogeneity, subsequent sensitivity analyses and meta-regression did not identify specific sources, and the pooled estimates remained stable. The use of a random-effects model further enhanced the robustness of our findings. The observed heterogeneity may partly reflect variability in continuous study-level characteristics, but this did not materially affect the overall conclusions, supporting their reliability and clinical relevance.

## Data Availability

The original contributions presented in the study are included in the article/Supplementary material, further inquiries can be directed to the corresponding author.

## Footnotes

^1^https://www.crd.york.ac.uk/PROSPERO/

## Supplementary material

SUPPLEMENTARY TABLE 1 The search strategy for PubMed.

SUPPLEMENTARY FIGURE 1 Model convergence and density plots. **(A)** Clinical Effective Rate (CER); **(B)** Cleveland Clinic Score (CCS).

SUPPLEMENTARY FIGURE 2 Risk of bias graph and summary.

SUPPLEMENTARY FIGURE 3 Comparison-adjusted funnel plot. **(A)** CER; **(B)** CCS.

SUPPLEMENTARY FIGURE 4 Node-splitting diagram for CER.

SUPPLEMENTARY FIGURE 5 Node-splitting diagram for CCS.

SUPPLEMENTARY FIGURE 6 Sensitivity analysis of CCS.

## References

[1] Su Y Zhang X Zeng J Pei Z Cheung RTF Zhou QP . New-onset constipation at acute stage after first stroke: incidence, risk factors, and impact on the stroke outcome. Stroke. (2009) 40:1304–9. doi: 10.1161/STROKEAHA.108.534776, PMID: 19228840

[2] Lim SF Childs C. A systematic review of the effectiveness of bowel management strategies for constipation in adults with stroke. Int J Nurs Stud. (2013) 50:1004–10. doi: 10.1016/j.ijnurstu.2012.12.002, PMID: 23279967

[3] Gao SG Zhao Y. Advances in post-stroke constipation research. J Liaoning Univ Tradit Chin Med. (2016) 18:142–5. doi: 10.13194/j.issn.1673-842x.2016.05.046

[4] Lu JY Nan H. Clinical observation of lactulose combined with microbial preparation in the treatment of stroke constipation. Modern Digest Intervent. (2017) 22:804–6.

[5] Colorectal and Anal Surgery Group, Chinese Society of Surgery, Chinese Medical Association . Guidelines for the diagnosis and treatment of chronic constipation in China. Chin J Gastroenterol. (2013) 18:605–12. doi: 10.3969/j.issn.1008-7125.2013.10.007

[6] Lindberg G Hamid SS Malfertheiner P Thomsen OO Fernandez LB Garisch J . World gastroenterology organisation global guideline: constipation--a global perspective. J Clin Gastroenterol. (2011) 45:483–7. doi: 10.1097/MCG.0b013e31820fb914, PMID: 21666546

[7] Wald A Scarpignato C Mueller-Lissner S Kamm MA Hinkel U Helfrich I . A multinational survey of prevalence and patterns of laxative use among adults with self-defined constipation. Aliment Pharmacol Ther. (2008) 28:917–30. doi: 10.1111/j.1365-2036.2008.03806.x, PMID: 18644012

[8] Page MJ McKenzie JE Bossuyt PM Boutron I Hoffmann TC Mulrow CD . The PRISMA 2020 statement: an updated guideline for reporting systematic reviews. BMJ. (2021) 372:n71. doi: 10.1136/bmj.n71, PMID: 33782057 PMC8005924

[9] Chinese Medical Association, Neurology Branch . 2016 edition of the Chinese guidelines and consensus on the management of cerebrovascular disease. Beijing: People's Health Publishing House. (2016) 106–150.

[10] Palsson OS Whitehead WE van Tilburg MA Chang L Chey W Crowell MD . Rome IV diagnostic questionnaires and tables for investigators and clinicians. Gastroenterology. (2016) 150:1481–91. doi: 10.1053/j.gastro.2016.02.014, PMID: 27144634

[11] Savović J Weeks L Sterne JA Turner L Altman DG Moher D . Evaluation of the Cochrane collaboration's tool for assessing the risk of bias in randomized trials: focus groups, online survey, proposed recommendations and their implementation. Syst Rev. (2014) 3:37. doi: 10.1186/2046-4053-3-37, PMID: 24731537 PMC4022341

[12] Mengersen KL Stojanovski E. Bayesian methods in meta-analysis chapter. (2006). doi: 10.3109/9781439822463.019

[13] Melsen WG Bootsma MC Rovers MM Bonten MJ. The effects of clinical and statistical heterogeneity on the predictive values of results from meta-analyses. Clin Microbiol Infect. (2014) 20:123–9. doi: 10.1111/1469-0691.12494, PMID: 24320992

[14] Seagroatt V Stratton I. Bias in meta-analysis detected by a simple, graphical test. Test had 10% false positive rate. BMJ. (1998) 316:470 author reply 470–1. doi: 10.1136/bmj.316.7129.469, PMID: 9492688 PMC2665628

[15] Salanti G . Indirect and mixed-treatment comparison, network, or multiple-treatments meta-analysis: many names, many benefits, many concerns for the next generation evidence synthesis tool. Res Synth Methods. (2012) 3:80–97. doi: 10.1002/jrsm.1037, PMID: 26062083

[16] Caldwell DM Ades AE Higgins JP. Simultaneous comparison of multiple treatments: combining direct and indirect evidence. BMJ. (2005) 331:897–900. doi: 10.1136/bmj.331.7521.897, PMID: 16223826 PMC1255806

[17] Jansen JP Naci H. Is network meta-analysis as valid as standard pairwise meta-analysis? It all depends on the distribution of effect modifiers. BMC Med. (2013) 11:159. doi: 10.1186/1741-7015-11-159, PMID: 23826681 PMC3707819

[18] Mavridis D Salanti G. A practical introduction to multivariate meta-analysis. Stat Methods Med Res. (2013) 22:133–58. doi: 10.1177/0962280211432219, PMID: 22275379

[19] Valkenhoef G. V. Kuiper J. Network meta-analysis using Bayesian methods (2015).

[20] Brooks S Gelman A. General methods for monitoring convergence of iterative simulations. J Comput Graph Stat. (1998) 7:434–55. doi: 10.1080/10618600.1998.10474787

[21] Page MJ Shamseer L Altman DG Tetzlaff J Sampson M Tricco AC . Epidemiology and reporting characteristics of systematic reviews of biomedical research: a cross-sectional study. PLoS Med. (2016) 13:e1002028. doi: 10.1371/journal.pmed.1002028, PMID: 27218655 PMC4878797

[22] Van Valkenhoef G Dias S Ades AE Welton NJ. Automated generation of node-splitting models for assessment of inconsistency in network meta-analysis. Res Synth Methods. (2016) 7:80–93. doi: 10.1002/jrsm.1167, PMID: 26461181 PMC5057346

[23] Stang A . Critical evaluation of the Newcastle-Ottawa scale for the assessment of the quality of nonrandomized studies in meta-analyses. Eur J Epidemiol. (2010) 25:603–5. doi: 10.1007/s10654-010-9491-z, PMID: 20652370

[24] Feng S Tang M Huang G Wang JM He S Liu D . EMG biofeedback combined with rehabilitation training may be the best physical therapy for improving upper limb motor function and relieving pain in patients with the post-stroke shoulder-hand syndrome: a Bayesian network meta-analysis. Front Neurol. (2023) 13:1056156. doi: 10.3389/fneur.2022.1056156, PMID: 36703623 PMC9873378

[25] Ma L Tang TT Zhang WW Yu H Shi BT. Observation on the efficacy of heat-sensitive moxibustion in treating post-stroke constipation with qi deficiency and its effect on serum SP and VIP levels. Shanghai J Acupunct Moxibustion. (2023) 42:251–5. doi: 10.13460/j.issn.1005-0957.2023.03.0251

[26] Chen YZ Wang YJ Xie ZG Guan SY. Clinical observation on bilateral acupoint acupuncture for the treatment of constipation in elderly stroke rehabilitation period. Hebei J Tradit Chin Med. (2022) 44:1007–10.

[27] Zhong Y Jia XM Wu YY. Clinical observation on the treatment of post-stroke constipation by acupuncture and regulating divine formulas. J Guangzhou Univ Tradit Chin Med. (2022) 39:1824–9. doi: 10.13359/j.cnki.gzxbtcm.2022.08.020

[28] Wang YP Li JC Ni YY Ji J Mu Y. Observation on the effect of integrated nursing care of combined Shangluo paste on Shenque acupoint in treating constipation after cerebral hemorrhage. J Navy Med. (2022) 43:531–4.

[29] Zhang JY Li ZM Zhang JH Yan WJ Yu MT. Observations on the efficacy of Xiao Yu Zi oil compresses on Shen que acupoints in patients with post-stroke gas constipation. J Shaanxi Univ Chin Med. (2022) 45:97–101. doi: 10.13424/j.cnki.jsctcm.2022.02.022

[30] Yuan XR . Clinical efficacy observation of Jin's three-needle therapy in treating post-stroke constipation. Guangzhou University of Chinese Medicine (2022). doi: 10.27044/d.cnki.ggzzu.2021.000136

[31] Xue G . Study on the effect of snap-needle therapy on patients with constipation in ischemic stroke with evidence of qi deficiency and blood stasis Changchun University of Chinese Medicine (2022). doi: 10.26980/d.cnki.gcczc.2021.000168

[32] Wang ZJ Liu CH Yang M Liu R. Treatment of post-stroke constipation by needling odd points combined with evidence-based point selection. Jilin J Tradit Chin Med. (2021) 41:119–22. doi: 10.13463/j.cnki.jlzyy.2021.01.032

[33] He XM Cai HL Liu YL Cai HR Xu JY Luo J . Observation on the efficacy of intermediate frequency therapy on constipation in patients with ischemic stroke. Clin J Chin Med. (2021) 13:110–2.

[34] Huang S Pan JZ. Recent efficacy of whole-body vibration training for the treatment of constipation after stroke in the elderly. Chinese Manipul Rehabil Med. (2021) 12:37–40. doi: 10.19787/j.issn.1008-1879.2021.12.014

[35] Song J . Clinical observation on the treatment of post-stroke constipation with Chinese medicine acupoint plasters. China's Naturopathy. (2021) 29:63–5. doi: 10.19621/j.cnki.11-3555/r.2021.0425

[36] Gao Y . Observation on the clinical efficacy of acupuncture point embedding in the treatment of post-stroke constipation with qi deficiency and its effect on serum SP and VIP levels Heilongjiang University of Chinese Medicine (2021). doi: 10.27127/d.cnki.ghlzu.2020.000101.36

[37] Du FF Wang XY Liu T. A study of the effect of deep muscle stimulator-assisted treatment of post-stroke constipation. Chin J Mod Drug Appl. (2020) 14:94–5. doi: 10.14164/j.cnki.cn11-5581/r.2020.11.043

[38] Cong N Zhang Y Guo C. Cognitive-behavioral intervention in stroke patients with constipation. China Med Herald. (2020) 17:189–92. doi: 10.20047/j.issn1673-7210.2020.34.048

[39] Liang BL Feng LY Zhang M Zeng XW Huang ZF. Observation on the nursing effect of abdominal massage method of "Fuyuan Tongzhi" on post-stroke constipation. J Clin Nurs. (2020) 19:79–81.

[40] Zhang H . Clinical study on the treatment of post-stroke constipation with deficiency evidence by laying moxibustion in the abdomen Henan University of Chinese Medicine (2022). doi: 10.27119/d.cnki.ghezc.2019.000065.40

[41] Sun XY . Clinical efficacy observation of acupuncture point embedding in the treatment of post-stroke constipation Anhui University of Chinese Medicine (2020). doi: 10.26922/d.cnki.ganzc.2019.000180.41

[42] Guo Q . Clinical efficacy observation on the treatment of post-stroke constipation by the method of burying the threads at the matching acupoints of Yuzhu Heilongjiang University of Chinese Medicine (2020). doi: 10.27127/d.cnki.ghlzu.2019.000140.42

[43] Wu L . Clinical efficacy of abdominal acupuncture in the treatment of constipation in patients with ischemic stroke Hunan University of Chinese Medicine (2020). doi: 10.27138/d.cnki.ghuzc.2019.000190.43

[44] Deng HM Tian Q Zeng KX. Clinical observation on the treatment of 29 cases of post-stroke constipation of qi deficiency and blood stasis type by burying threads. Hunan J Tradit Chin Med. (2019) 35:64–5. doi: 10.16808/j.cnki.issn1003-7705.2019.01.030

[45] Huang LY . Observations on the clinical efficacy of Baresha point on constipation after stroke Guangzhou University of Chinese Medicine (2019).

[46] Guan FY . Clinical study on the treatment of post-stroke constipation with acupuncture point embedding Guangzhou University of Chinese Medicine (2019).

[47] Li YQ Wang LQ Guo C Jiang YH Sun XW Dong X . Study on the effect of burying threads at the Tianshu point combined with the foot transportation sense area of the head on serum SP and VIP levels in patients with post-stroke constipation. J Clin Acupunct Moxibust. (2018) 34:50–4.

[48] Li G Wang YB Zhao XH Cai YY Wu Y Yang J . Observation on the recent efficacy and recurrence rate of gastrointestinal intermediate frequency therapeutic instrument-assisted lactulose in the treatment of constipation secondary to cerebral infarction. J Clin Exp Med. (2018) 17:60–3.

[49] Ma YM Chen WG Jiang NN Guo AS Xu Q Hu YM . The efficacy of biofeedback in the treatment of post-stroke constipation. Chin J Rehabil Med. (2018) 33:585–7.

[50] Zhao JJ . Clinical study on the prevention of post-stroke constipation after stroke by applying the umbilical cord method with wu juju Guangzhou University of Chinese Medicine (2018).

[51] Long XN Liu LM. Observation on the efficacy of auricular acupuncture point burying beans in the treatment of post-stroke constipation. J Anhui Tradit Chinese Med College. (2017) 36:50–2.

[52] Li H Kuang SR Yu XF. Observation on the effect of abdominal acupoint massage with electric massager in preventing and treating constipation in patients with cerebral hemorrhage. Chin Nurs Res. (2017) 31:972–4.

[53] Yu HL . Clinical efficacy observation of Shenque acupoint patch method based on disease mechanism on patients with constipation during stroke recovery period Beijing University of Chinese Medicine (2017).

[54] Liu YT . Clinical study on the treatment of post-stroke constipation by the method of "heavy moxibustion" with interspersed ginger paste Henan University of Chinese Medicine (2017).

[55] Peng YJ Sun JH Li ZR. Clinical observation on the treatment of post-stroke constipation by deep stabbing Tianshu point with electroacupuncture. Shanghai J Acupunct Moxibust. (2016) 35:1181–3. doi: 10.13460/j.issn.1005-0957.2016.10.1181

[56] Ji J Wang YW Ren SL. The efficacy of auricular acupressure with basic nursing care in the treatment of solid constipation in the acute phase of stroke. Shanghai J Acupunct Moxibust. (2016) 35:276–8. doi: 10.13460/j.issn.1005-0957.2016.03.0276

[57] Chen Y . Analysis of the effect of combined abdominal massage and acupressure care on defecation in elderly stroke patients. Chin J Geriatr Care. (2016) 14:110–1.

[58] Ma LP Yang CL Zhong GY. Effect of cognitive-behavioral intervention on the outcome of stroke patients with constipation. Chin J Gerontol. (2016) 36:420–2.

[59] Luo YY He Y Deng M. Study on the improvement of symptoms and cognitive ability of patients with constipation after stroke by nursing intervention. J Colorectal Anal Surg. (2016) 22:220–4.

[60] Tu XH Deng YD Liang CQ Ma YL Zheng MY. Nursing intervention for constipation and cognitive function after stroke. Chinese J Hygiene Rescue. (2016) 2:190–3.

[61] Wang B Chen YW Guo JJ. Analysis of the efficacy of traditional Chinese medicine acupoint patch in the treatment of post-stroke constipation. Nei Mongol J Tradit Chin Med. (2016) 35:106. doi: 10.16040/j.cnki.cn15-1101.2016.13.107

[62] Zhang B . Clinical observation on the treatment of post-stroke constipation by burying beans in ear acupuncture points Anhui University of Chinese Medicine (2016).

[63] Lv LM . Clinical study of auricular magnetic bead pressing intervention for constipation in stroke patients Fujian University of Traditional Chinese Medicine (2016).

[64] Ma Y Li C. Clinical observation of thunder fire moxibustion in the treatment of qi deficiency type constipation during post-stroke period. Chinese Med Modern Distance Educ China. (2015) 13:73–5.

[65] Liu CM Feng XD Liu FL Wang XD Wang LN Niu YL . Efficacy of navel moxibustion in the treatment of post-stroke constipation. Chin J Rehabil Theory Pract. (2015) 21:1209–11.

[66] Wang Q . Observation on the nursing effect of auricular burying seeds on constipation in patients with cerebral infarction. Nei Mongol J Tradit Chin Med. (2015) 34:175–6.

[67] Zhu WX . Clinical study of moxibustion of Shenque acupoints across onion and black onion cakes in the treatment of post-stroke constipation Guangzhou University of Chinese Medicine (2015).

[68] Huang P Li SL. Clinical effects of nursing interventions for cognitive function in patients with constipation after stroke. World Chin J Dig. (2014) 22:3166–9. doi: 10.11569/wcjd.v22.i21.3166

[69] Chu JM Bao YH Li LP Lin L. Clinical observation on prevention of post-stroke constipation by heat-sensitive moxibustion. Chin Arch Tradit Chin Med. (2013) 31:217–9. doi: 10.13193/j.archtcm.2013.01.221.chujm.034

[70] Xu HM Liu CX Wang CX. Observation on the efficacy of auricular pressure bean in the treatment of constipation in stroke. Clin J Tradit Chin Med. (2013) 25:1003–4. doi: 10.16448/j.cjtcm.2013.11.014

[71] Reng Z Wu QM Li DD Liu WA Li XR Lin XM . Treatment of post-stroke constipation with acupuncture by regulating qi and ventilating internal organs. Chin Acupunct Moxibust. (2013) 33:893–6. 24377218

[72] Zhuang WR . Observation on the therapeutic effect of Chinese medicine acupoint dressing in treating post-stroke constipation Guangzhou University of Chinese Medicine (2012).

[73] Zhao Y . Therapeutic efficacy of combining head and body acupuncture in the treatment of constipation after ischemic stroke. Shanghai J Acupunct Moxibust. (2010) 29:436–8.

[74] Zhao JL Zhang B Huang JH Chen RX. Clinical observation on the treatment of constipation after ischemic stroke by thermal moxibustion. Liaoning J Tradit Chin Med. (2010) 37:1114–5. doi: 10.13192/j.ljtcm.2010.06.159.zhaojl.044.74

[75] Meng ZX . Clinical observation on Renmai moxibustion for treatment of post-stroke constipation Shandong University of Chinese Medicine (2023). doi: 10.27282/d.cnki.gsdzu.2023.001013.75

[76] Han YF . Clinical observation of acupuncture in the treatment of constipation after stroke of qi deficiency based on the theory of "circulation of one qi" Liaoning University of Chinese Medicine (2023). doi: 10.27213/d.cnki.glnzc.2023.000301.76

[77] Han WH Zhan LF Jiang J Yan TY Zhu MH Chen Y . Clinical efficacy observation of acupuncture based on the theory of 'regulating pivot and promoting stomach function' on constipation after stroke of blood deficiency and intestinal dryness type. China J Tradit Chin Med Pharm. (2024) 39:3221–6.

[78] Lumley T . Network meta-analysis for indirect treatment comparisons. Stat Med. (2002) 21:2313–24. doi: 10.1002/sim.1201, PMID: 12210616

[79] Camilleri M . Serotonin in the gastrointestinal tract. Curr Opin Endocrinol Diabetes Obes. (2009) 16:53–9. doi: 10.1097/MED.0b013e32831e9c8e, PMID: 19115522 PMC2694720

[80] Jiang JZ Liu ZC. Advances in clinical and mechanistic studies of acupuncture point embedding therapy. J Liaoning Univ Tradit Chin Med. (2009) 11:31–4.

[81] Zhang SY Gong XH Ji CH Xuan LH Zhang LM Zhou QR . Observations on the efficacy of simple acupoint burrowing method in the treatment of habitual constipation. Chin Arch Tradit Chin Med. (2012) 30:1286–8.

[82] Ma YQ . Chinese embedded thread therapy guide. Beijing: Chinese Medicine Science and Technology Publishing House (1994). 6 p.

[83] Wen MS . An experimental study on the integrative effect and therapeutic mechanism of acupoint embedding therapy. J Shaanxi Univ Chinese Med. (1993) 11:6–7.

[84] Huo J Zhao JQ Yuan Y Wang J. Current status of research on the mechanism of action of acupoint buried thread therapy. Zhongguo Zhen Jiu. (2017) 37:1251–4. doi: 10.13703/j.0255-2930.2017.11.031, PMID: 29354967

[85] Du J Liu H Xu J. Treatment of post-stroke constipation by acupoint burrowing: a multicenter randomized controlled study. Chin Acupunct Moxibust. (2020) 40:493–7. 10.13703/j.0255-2930.20190507-k0004 32394656

[86] Tang, OF Huang SM Ye XX. Net meta-analysis of the efficacy of acupuncture for constipation in stroke patients. Chin Acupunct Moxibust. (2020) 40:1011–6. 10.13703/j.0255-2930.20190717-k0008 32959600

[87] Wang XL Lin GH Xu N Zeng JC Xu DH Wang SX . Analysis of reports of adverse reactions to acupuncture point burrowing. Chin Acupunct Moxibust. (2020) 40:193–6. +210 10.13703/j.0255-2930.20190316-k00034 32100507

[88] Zhou JY Wang J Ning BF Hu YD Zhao Q Tan W . Sustained ameliorating effects and autonomic mechanisms of transcutaneous electrical acustimulation at ST36 in patients with chronic constipation. Front Neurosci. (2022) 16:1038922. doi: 10.3389/fnins.2022.1038922, PMID: 36478881 PMC9720110

[89] Ueno N Inui A Satoh Y. The effect of mosapride citrate on constipation in patients with diabetes. Diabetes Res Clin Pract. (2010) 87:27–32. doi: 10.1016/j.diabres.2009.09.024, PMID: 19889470

[90] Margolis KG Cryan JF Mayer EA. The microbiota-gut-brain Axis: from motility to mood. Gastroenterology. (2021) 160:1486–501. doi: 10.1053/j.gastro.2020.10.066, PMID: 33493503 PMC8634751

[91] Sun R Zhang J Liu JL Zeng JC Xu DH Wang SX . Effects of moxibustion at Shenque acupoint on clinical symptoms and intestinal flora in patients with post-stroke constipation. Lishizhen Med Mater Res. (2023) 34:1414–6.

[92] Yang HY Hu ZC. Biophysical properties of moxibustion. Chin Acupunct Moxibust. (2009) 29:897–9. 19994689

[93] Wang LL . Characteristics and warming effect of moxibustion. Chin Acupunct Moxibust. (2011) 31:865–8. 22043667

[94] Chang XR Peng L Yi SX Peng Y Yan J. Association of high expression in rat gastric mucosal heat shock protein 70 induced by moxibustion pretreatment with protection against stress injury. World J Gastroenterol. (2007) 13:4355–9. doi: 10.3748/wjg.v13.i32.4355, PMID: 17708611 PMC4250864

[95] Zhang C Xu Z Huang D Zhang LQ Zou J Wang Y . Collagen fiber content in acupoint and non-meridian non-acupoint areas. J Guiyang College Trad Chinese Med. (2018) 40:16–21.

[96] Liu X Guo XC Lin YY Xu YH Wang YP Wu CB . A study of the biophysical properties of skin at acupoints and non-acupoints as affecting the permeation properties of mustardine. Chin Herbal Med. (2013) 44:1111–6.

[97] Joseph J Depp C Shih PB Cadenhead KS Schmid-Schönbein G. Modified Mediterranean diet for enrichment of short chain fatty acids: potential adjunctive therapeutic to target immune and metabolic dysfunction in schizophrenia? Front Neurosci. (2017) 11:155. doi: 10.3389/fnins.2017.00155, PMID: 28396623 PMC5366345

[98] Liu CL Wang XH Wang F Liu F Dang R Feng ZJ . A study on the distribution of trace elements in acupoints. Zhongguo Zhong Yi Ji Chu Yi Xue Za Zhi. (2016) 22:1215–8.

