# Supplementary material for: Comparative effectiveness of multiple different non-pharmacologic interventions for post-stroke constipation: a Bayesian network meta-analysis
Source: Front Neurol. 2025 Oct 10;16:1591620. doi: 10.3389/fneur.2025.1591620 (PMC12551397; doi:10.3389/fneur.2025.1591620)
Supplement: SUPPLEMENTARY TABLE 1 — The search strategy for PubMed. [file Table_1.docx]

Supplementary Material Table 1. The search strategy for PubMed.

| Number | Search terms |
| --- | --- |
| #1 | “Stroke” [Mesh] OR “Strokes” [Title/Abstract] OR “Cerebrovascular Accident” [Title/Abstract] OR “Cerebrovascular Stroke” [Title/Abstract] OR “Apoplexy” [Title/Abstract] OR “Cerebral Stroke” [Title/Abstract]OR “Vascular Accidents, Brain” [Title/Abstract] OR “CAV” [Title/Abstract] OR “cerebral infarction” [Title/Abstract] OR “Ischemic Stroke” [Title/Abstract] OR “cerebral hemorrhage [Title/Abstract] OR “Hemorrhage Stroke” [Title/Abstract] |
| #2 | “Constipation” [Mesh] OR “Dyschezia” [Title/Abstract] OR “Colonic Inertia” [Title/Abstract] |
| #3 | “Acupuncture Therapy” [Mesh] OR “Acupuncture” [Mesh/Title] OR “Acupoint Catgut Embedding” [Title/Abstract] OR “Moxibustion” [Mesh] OR “Acupuncture, Ear” [Mesh] OR “Auricular Therapy ” [Title/Abstract] OR “Auricular Point Pressing” [Title/Abstract] OR “massage” [Mesh] OR “Abdominal massage” [Title/Abstract] OR “Point Application” [Title/Abstract] OR “Acupoint Application Therapy” [Title/Abstract] OR “Physical Therapy Modalities” [Mesh] OR “Physical Therapy” [Title/Abstract] OR “Physiotherapy” [Title/Abstract] OR “Rehabilitation”[Mesh] OR “Rehabilitation Training” [Title/Abstract] OR “Cognitive Behavioral Training ” [Title/Abstract] |
| #4 | “Randomized Controlled Trial[Publication Type]OR “RCT randomized controlled”[Publication Type] OR “random allocation”[Title/Abstract] OR “allocation, random”[Title/Abstract] OR “randomized, controlled”[Title/Abstract] OR “clinical trial”[Title/Abstract] |
| #6 | #1 AND #2 AND #3 AND #4 |
