# Supplementary figures and images for: Comparative effectiveness of multiple different non-pharmacologic interventions for post-stroke constipation: a Bayesian network meta-analysis
Source: Front Neurol. 2025 Oct 10;16:1591620. doi: 10.3389/fneur.2025.1591620 (PMC12551397; doi:10.3389/fneur.2025.1591620)

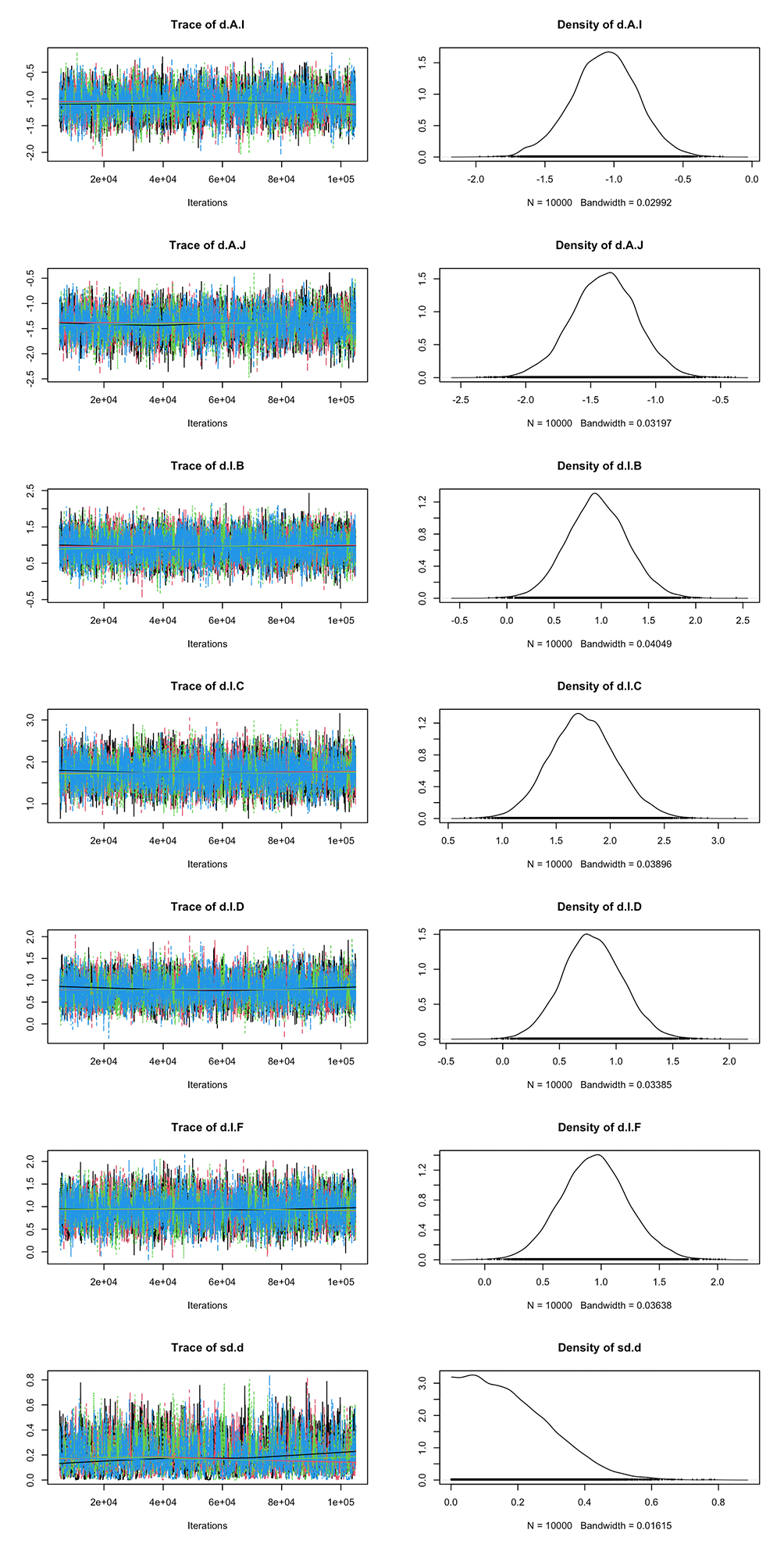

Supplement: SUPPLEMENTARY FIGURE 1 — Model convergence and density plots. (A) Clinical Effective Rate (CER); (B) Cleveland Clinic Score (CCS). [file Image_1.TIF]

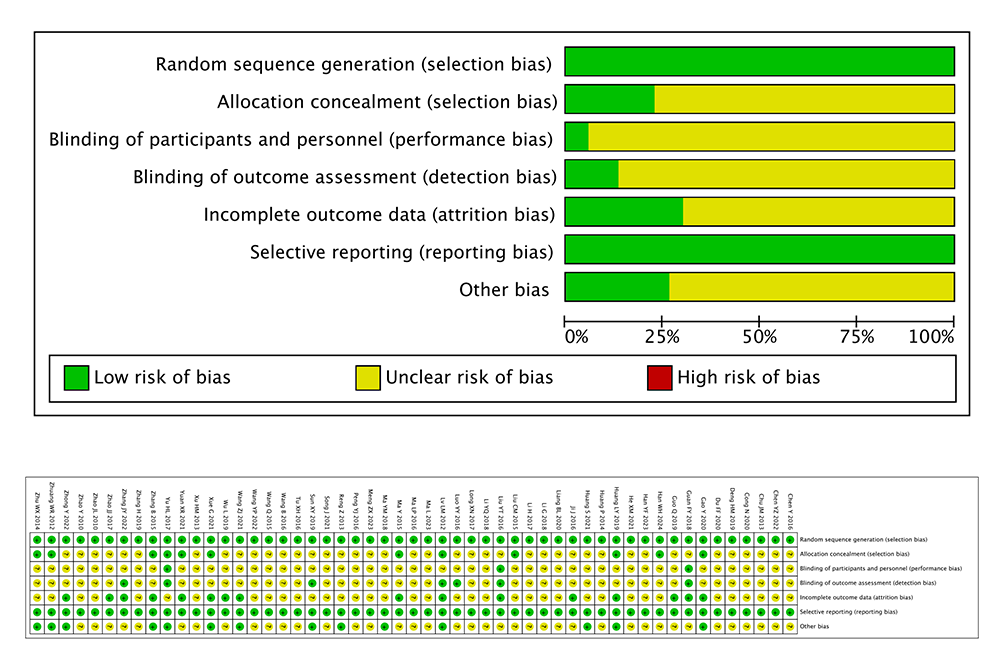

Supplement: SUPPLEMENTARY FIGURE 2 — Risk of bias graph and summary. [file Image_2.TIF]

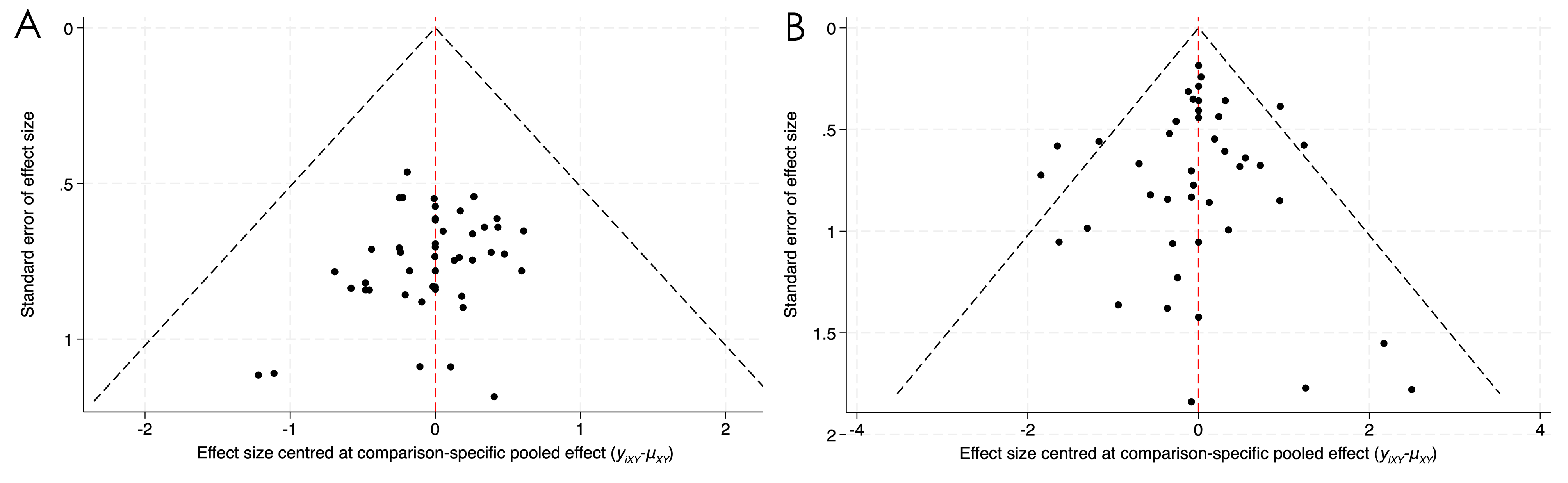

Supplement: SUPPLEMENTARY FIGURE 3 — Comparison-adjusted funnel plot. (A) CER; (B) CCS. [file Image_3.TIF]

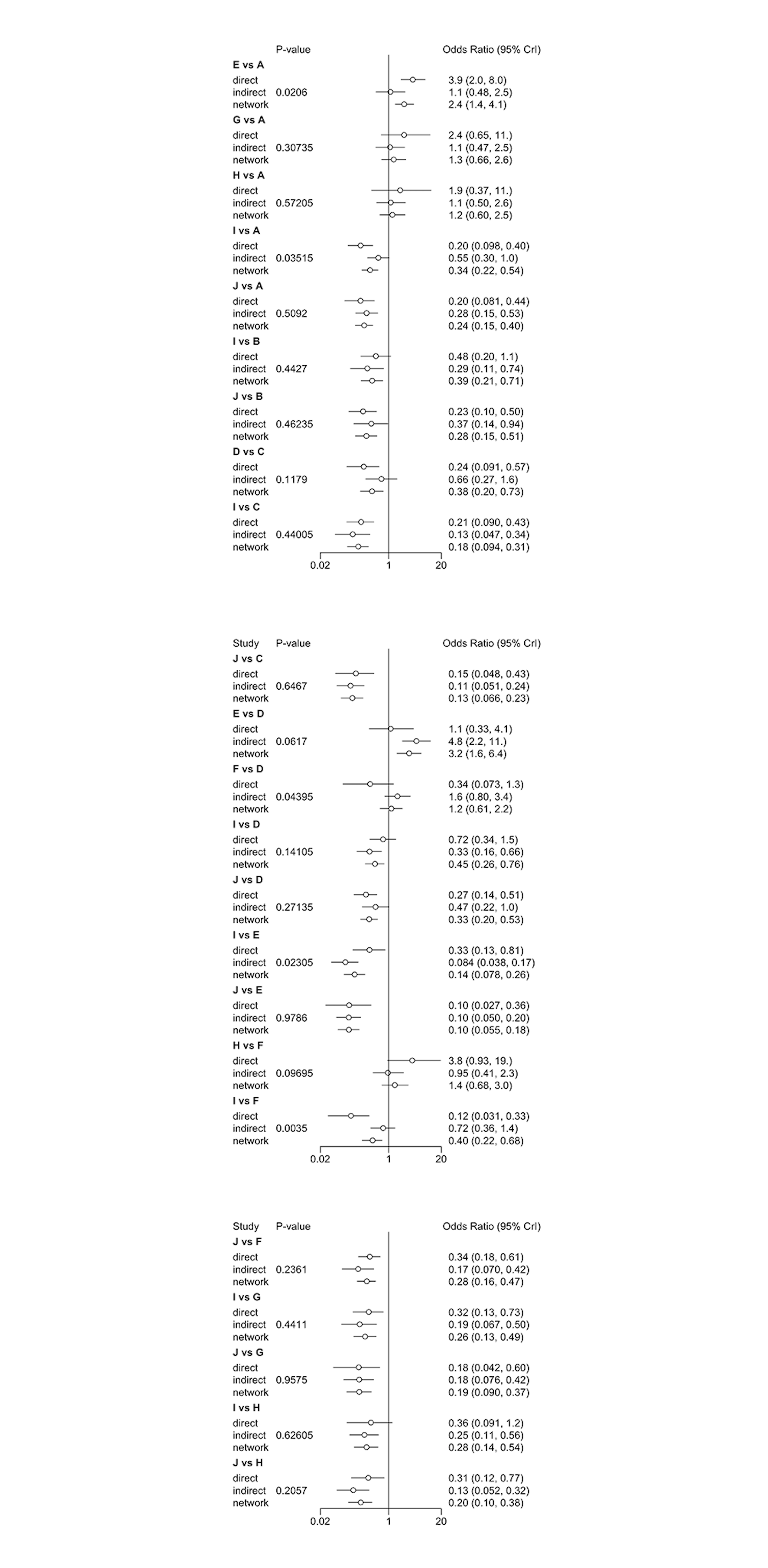

Supplement: SUPPLEMENTARY FIGURE 4 — Node-splitting diagram for CER. [file Image_4.TIF]

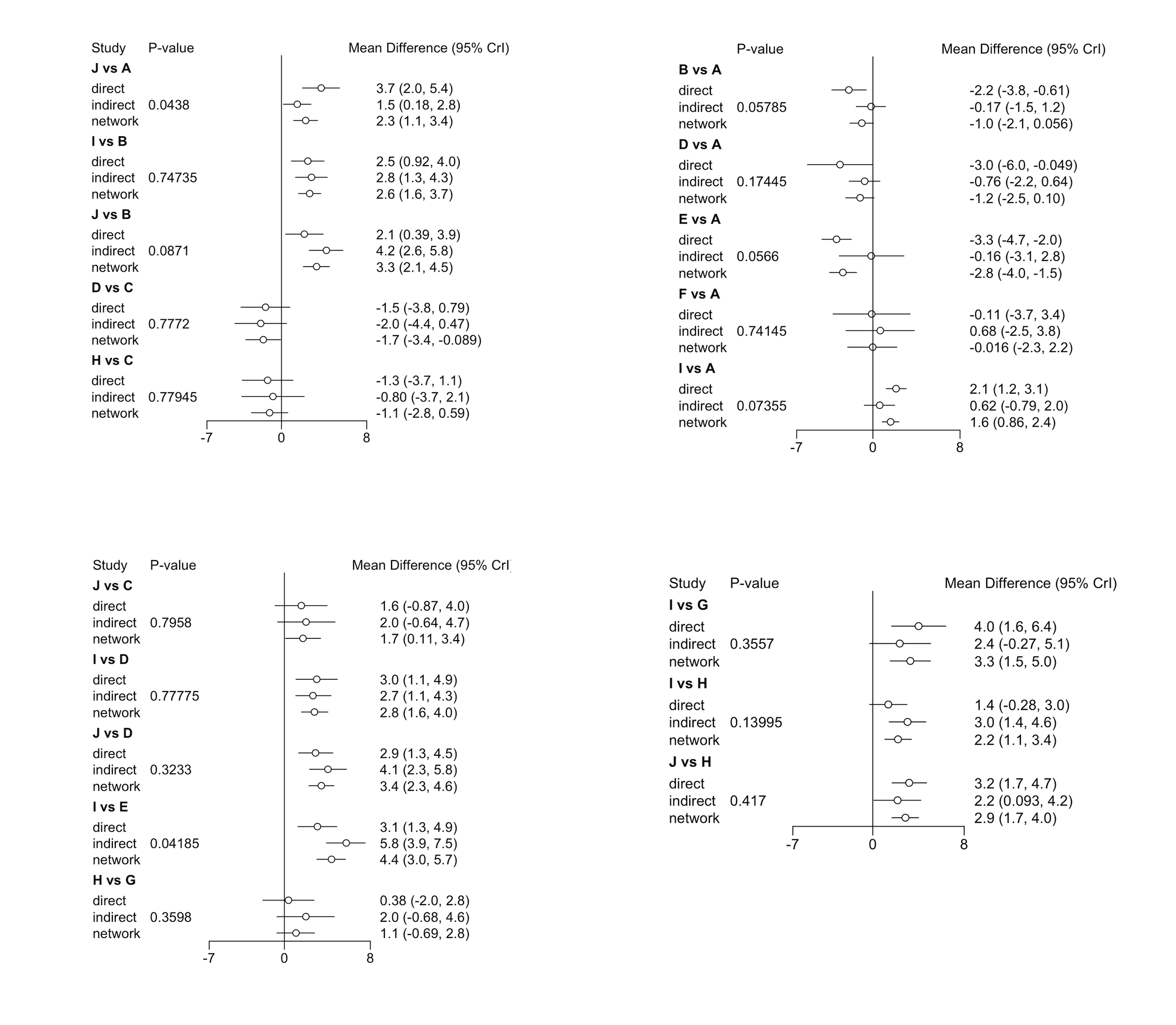

Supplement: SUPPLEMENTARY FIGURE 5 — Node-splitting diagram for CCS. [file Image_5.TIF]

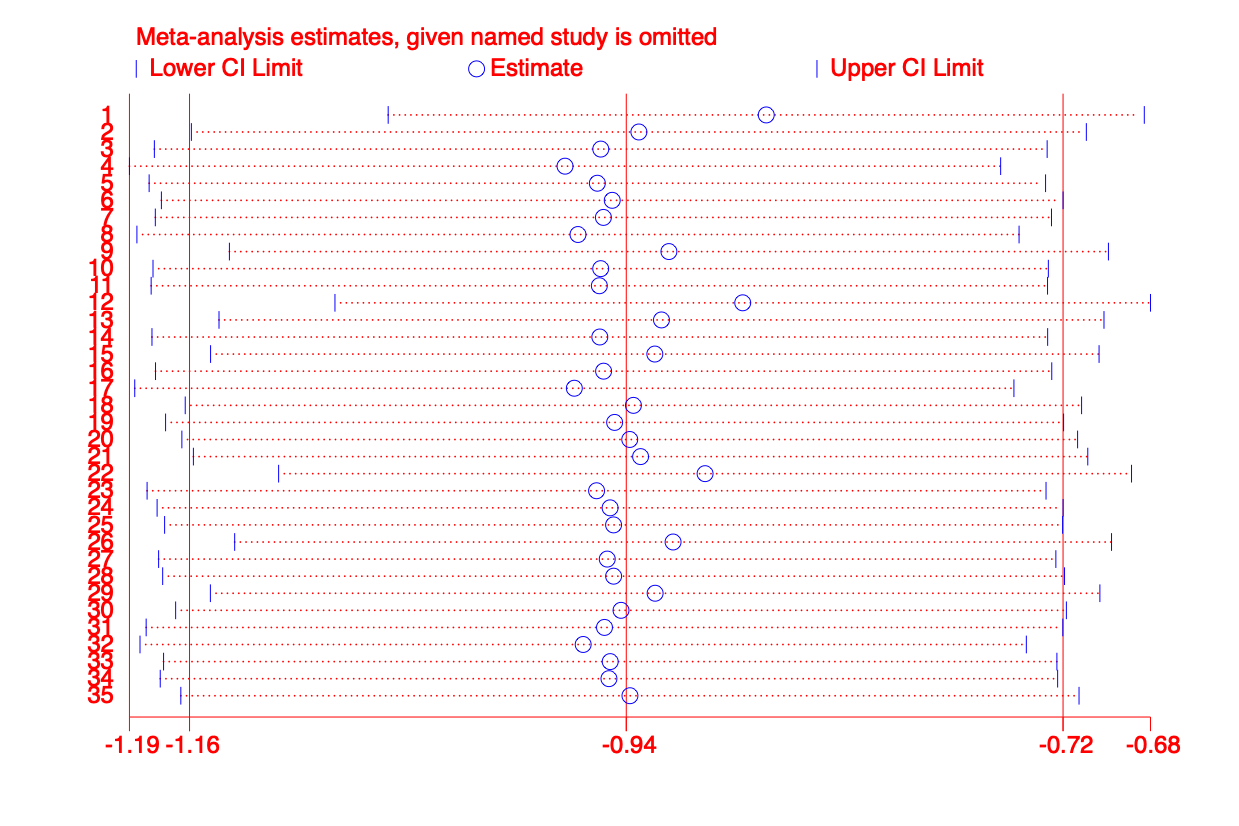

Supplement: SUPPLEMENTARY FIGURE 6 — Sensitivity analysis of CCS. [file Image_6.TIF]
