# Supplementary material for: Comparative effectiveness of multiple different non-pharmacologic interventions for post-stroke constipation: a Bayesian network meta-analysis
Source: Front Neurol. 2025 Oct 10;16:1591620. doi: 10.3389/fneur.2025.1591620 (PMC12551397; doi:10.3389/fneur.2025.1591620)
Supplement: Supplementary file 8 [file Table_2.docx]

Supplementary Material Table 2. Egger's test for CER

Std_Eff | Coef. Std. Err. t P>|t| [95% Conf. Interval]

slope | 1.221346 .3396244 3.60 0.001 .5349395 1.907753

bias | -.2105969 .1196325 -1.76 **0.086** -.4523831 .0311894
