# Supplementary material for: Comparative effectiveness of multiple different non-pharmacologic interventions for post-stroke constipation: a Bayesian network meta-analysis
Source: Front Neurol. 2025 Oct 10;16:1591620. doi: 10.3389/fneur.2025.1591620 (PMC12551397; doi:10.3389/fneur.2025.1591620)
Supplement: Supplementary file 9 [file Table_3.docx]

Supplementary Material Table 3. Egger's test for CCS

Std_Eff | Coef. Std. Err. t P>|t| [95% Conf. Interval]

slope | -2.293662 .4236797 -5.41 0.000 -3.155645 -1.43168

bias | -.2842176 .8215843 -0.35 **0.732** -1.955743 1.387308
