# Supplementary material for: Comparative effectiveness of multiple different non-pharmacologic interventions for post-stroke constipation: a Bayesian network meta-analysis
Source: Front Neurol. 2025 Oct 10;16:1591620. doi: 10.3389/fneur.2025.1591620 (PMC12551397; doi:10.3389/fneur.2025.1591620)
Supplement: Supplementary file 10 [file Table_4.docx]

Supplementary Material Table 5. Meta-regression for CCS

_ES | Coef. Std. Err. t P>|t| [95% Conf. Interval]

year | .0969034 .3286507 0.29 0.770 -.5733841 .7671908

time | .2216998 .4061212 0.55 0.589 -.60659 1.049989

control subjects | .2242482 .3217069 0.70 0.491 -.4318774 .8803737

_cons | -3.529896 1.080198 -3.27 0.003 -5.732974 -1.326817
